# An Advanced Undergraduate Laboratory Course: Calcium Imaging and Data Analysis – a Mini Graduate Research Experience

**DOI:** 10.59390/001c.154223

**Published:** 2025-12-31

**Authors:** Hua Bai, Zhuo Fu, Lina Ni

**Affiliations:** 1 School of Neuroscience Virginia Tech

**Keywords:** calcium imaging, calcium imaging analysis, python, ImageJ, TrackMate, lab activity

## Abstract

Calcium imaging has become a powerful and widely used technique to visualize and measure neural activity in real time, offering valuable insights into complex behaviors, neural networks, and brain function. However, implementing calcium imaging in undergraduate laboratories is challenging due to the need for specialized microscopy and advanced computational skills for data analysis. To help undergraduate students grasp the principles and analytical methods of calcium imaging through hands-on experience, we designed and taught a semester-long course. The course is structured around four key components, which reflect essential elements of graduate research training. (1) AI-assisted literature review helps students understand the fundamental principles of calcium imaging, its analytical methods, and relevant background information. (2) Calcium imaging demonstration provides students with hands-on exposure to calcium imaging procedures in a research setting. (3) Calcium imaging analysis introduces students using Python to analyze real research calcium imaging datasets. (4) Poster presentation allows students to present their findings and strengthen their scientific communication skills. The pedagogical goals of this course are to enhance students’ understanding of calcium imaging and its analytic methods while strengthening their abilities to read scientific literature and effectively communicate their research. Progress toward these goals is assessed through multiple approaches, including chalk talks, written assignments, code-based data analyses, and poster presentations. In addition, this course mirrors real research process to help students explore potential career paths in science.

Calcium imaging has evolved into an important technique in neuroscience research, offering a precise method for assessing neuronal excitability through intracellular calcium concentration (Grienberger & Konnerth, 2012; Russell, 2011). Calcium imaging relies on calcium indicators to measure intracellular calcium levels to estimate neural activity. Two types of calcium indicators are commonly used: chemical indicators and genetically encoded indicators. When intracellular calcium levels increase, calcium binds to and induces structural changes of calcium indicators that increase their fluorescence intensity (Grienberger & Konnerth, 2012). Thus, calcium imaging enables real-time visualization and measurement of neural activity, allowing researchers to monitor both individual neurons and large neuronal populations in freely behaving animals, thereby providing valuable insights into complex behaviors, its associated neural networks, and the brain’s function (Oh et al., 2019).

Implementing calcium imaging in undergraduate laboratories can be very challenging. Performing calcium imaging often requires access to specialized microscopy (i.e., confocal or two-photon microscopes), that are expensive and technically challenging to operate. These instruments are typically reserved for research rather than teaching, which limits undergraduate access, impedes both conceptual and procedural learning, and exacerbates academic achievement gaps in the educational pipeline. In addition, calcium imaging produces large, high-dimensional datasets that are consistent with “big data” analytics of the modern day. The analysis of calcium imaging datasets requires both biological knowledge of neuronal physiology and computational skills in image processing and quantitative data analysis, often involving using programming languages such as Python (Robbins et al., 2021).

*Drosophila* larval warm cells offer an excellent and affordable model system for undergraduate students to understand the fundamental principles of calcium imaging and data analysis. Each side of the larval head contains two warm cells. These cells are activated by warming when larvae are submerged in buffer solutions (Hernandez-Nunez et al., 2021; Omelchenko, Bai, Spina, et al., 2022). In this lab, we used the GAL4/UAS system to express the genetically encoded calcium indicator GCaMP6m in warm cells by *Ir68a-Gal4* (Brand & Perrimon, 1993; Chen et al., 2013; Hernandez-Nunez et al., 2021). The small number of neurons, minimal background noise, and robust activation upon warming make it practical for students to visualize and quantify calcium responses. We use confocal microscopy to perform calcium imaging that captures not only x/y movement but also the motion along the z-axis for three-dimensional (3D) reconstruction and subsequent analyses (Omelchenko, Bai, Hussain, et al., 2022). This experience enables students to understand the 3D nature of z-stack imaging and the challenges posed by movement in 3D during live imaging experiments.

This semester-long course was designed to introduce undergraduate students to the principles and analytical methods of calcium imaging through rich hands-on experiences. The curriculum was structured around four key modules: 1) literature review; 2) calcium imaging demonstration; 3) data analysis; and 4) poster presentation. During the literature review, students explored foundational concepts in calcium imaging, including its principles, analytical methods, and relevant scientific context. This component aimed to establish a foundational understanding of the hands-on work that followed. The calcium imaging demonstration was performed using the Nikon SoRa Spinning Disk Microscope. It provided students with hands-on exposure to calcium imaging techniques in a research setting, thereby enhancing their understanding of experimental procedures and data acquisition. The calcium imaging analysis introduced students to using Python to process and analyze calcium imaging datasets. This component emphasized the development of computational skills essential for interpreting imaging data. At the end of the course, students synthesized their findings into a scientific poster, presenting their work to their peers and instructors. This learning experience was designed to strengthen their scientific communication and presentation skills. The pedagogical objectives of this course included understanding the concept of calcium imaging, employing the GAL4/UAS system to express genetically encoded calcium indicators in specific cells, using confocal microscopy to conduct calcium imaging, and developing computational skills to analyze and interpret calcium imaging data. In addition, students created and presented scientific posters to strengthen their communication skills. Beyond technical competencies, the course aimed to simulate authentic research laboratories to encourage students to further explore potential career paths in science.

## MATERIALS AND METHODS

### *Drosophila* Strains and Maintenance

The fly strains used in the calcium imaging experiments were *UAS-GCaMP6m* (BDSC 42750) and *Ir68a-Gal4* (Knecht et al., 2017). Flies were maintained at 25 °C in standard cornmeal medium under a 12 h:12 h light: dark cycle with 50% relative humidity (Pervical, DR-41VL). The cornmeal medium was prepared with the following ingredients: 79 g/L D-(+)-glucose (Genesee, 62-113), 7.5 g/L agar (Genesee, 62-111), 24 g/L flaked yeast (Genesee, 62-108), 57 g/L cornmeal (Genesee, 62-110), 2.1 g/L methyl 4-hydroxybenzoate (Thermo Scientific, 126965000) (dissolved in 11.1 mL of 95% ethanol (Decon Labs, Inc., 64-17-5)), 6 g/L sodium potassium tartrate tetrahydrate (Thermo Scientific, 033241-36), and 0.9 g/L calcium chloride (Fisher Scientific, 10035-04-8) in 1 L of distilled water. The mixture was boiled to dissolve all ingredients and dispensed into fly vials (Genesee, 32-113RL and 49-102) before cooling and solidification (Huda et al., 2025).

### Calcium Imaging

Fly larvae preparation and calcium imaging were performed as described (Omelchenko, Bai, Hussain, et al., 2022) with minor modifications. For sample preparation, flies were briefly anesthetized with CO_2_ (Airgas, UN1013), sorted into vials with 20-30 males and 20-30 females each, and allowed to recover for 2-3 days. To synchronize larval age, flies were transferred to fresh vials with yeast granules (Red Star) and allowed to lay eggs for 6-8 hours before removal. After 72 hours, early third-instar larvae were collected using a 20% sucrose solution (Fisher Scientific, 220-212) and imaged within one hour of collection.

For imaging, a single larva was placed at the center of a slide (Fisher Scientific, 12-544-2), submerged in 1 x PBS (Thermo Fisher Scientific, 10010-031), and covered with a cover glass (VWR, 48382-126) to immobilize the animal. Then, the sample slide was placed upside down on the stage of a Nikon SoRa Spinning Disk Microscope (Yokogawa CSU-W1 SoRa with 50 μm disks, two Orca FusionBT sCMOS cameras built around an inverted Nikon Ti2 microscope, Queensgate piezo stage with a 450 μm range, 20 x air objective, 488 nm laser excitation). Imaging settings included: 656 pad; 16-bit format; 115 ms exposure; full sensor resolution (2304 x 2304); 35% fluorescence intensity; 1 x zoom; 2.8 x SoRa magnifier; z-stack with 21 slices over 30-40 μm z-distance; and triggered NIDAQ Piezo Z acquisition. A temperature probe (Physitemp, IT-24P) was gently taped into position so that its tip rested near the larva, and a cooling Peltier device (Peltier cooling module: TE Technology, TE-127-1.0-0.8; heat sink: SGTKJSJS, 2DSRP-1571; power supply: Circuit SpecialistsCSI1802X) was placed on top of the slide. Calcium imaging data acquisition and temperature recording (thermocouple data acquisition device: Measurement Computing, USB-2001-TC; DAQami software: Measurement Computing) were started simultaneously. The temperature stimulus protocol started with 30 seconds at room temperature (~ 25 °C), followed by 30 seconds of cooling to ~ 10 °C, and 30 seconds of warming back to ~ 25 °C. The cooling and warming cycle was repeated three times.

Fly maintenance and calcium imaging were performed by instructors.

### Calcium Imaging Analysis

Detailed instructions, example datasets, and scripts were provided in a GitHub repository ( https://github.com/hbai521/calcium_imaging/).

#### Preparation and Software Setup

Fiji (https://fiji.sc/) (Schindelin et al., 2012) and Python (https://www.python.org/) were downloaded and installed. A Python IDE (JetBrains, PyCharm, 2025) was used in class to run the analysis scripts. Three Python packages, Pandas, Matplotlib, and NumPy, were installed using pip or a requirements.txt file (GitHub: Requirements and Preparation and Setup Instructions).

#### Extraction of Fluorescence Intensities Using TrackMate in ImageJ

The ND2 files were exported from NIS-Elements using the following settings: data were saved as multiple TIFF files, with each file containing “t” dimensions, monochrome image per channel, preserved bit depth, and no LUTs applied. This process generated 21 TIFF files, each representing a single z-slice with 88 time points. The TIFFs were then opened in Fiji for processing.

The analysis began by selecting a z-stack slice that exhibited visible fluorescence signals across all time points (GitHub: Workflow > Step 1: Visualizing Neuron Response in ImageJ). TrackMate was then used to extract fluorescence intensity over time using the following settings: Difference of Gaussian (DoG) detector, blob diameter set between 22-36 pixels (based on neuronal shortest diameter), adjusted quality threshold to capture weaker signals, sub-pixel localization, median filtering, and spatial (x and y) filters to isolate the target neuron. Intensity data were exported from the “All Spots Table” as CSV files, named according to the corresponding z-slice (e.g., data from the fourth z-slice were saved as Mean_Intensity04.csv) (GitHub: Workflow > Step 2: Extracting Fluorescence Intensity Using TrackMate). Prior to analysis, extra headers, incorrect tracks, and duplicate entries were removed. Background fluorescence was measured at five time points from nearby non-neuronal regions using Fiji’s Measure tool and recorded in a file named background_i.csv (GitHub: Workflow > Step 3: Background Correction). This process was repeated for all z-slices in which the neuron was visible, and all CSV files for each neuron were saved in the same folder (GitHub: Workflow > Step 4: Work on Additional Z-stacks).

#### Using Python to Extract Maximum Intensities Across Time

After fluorescence intensities were extracted, Python was used to compute the calcium changes over time by extracting the maximum signal across all z-slices for each time point. A script (CIAnalysis_120s.py) iterated through all CSV files for each neuron, extracted the highest fluorescence value at each time-point (POSITION_T), and incorporated background values (recorded in background_i.csv) to calculate the normalized fluorescence change as ΔF/F_min_. The output was a merged dataset (merged_data.csv) and the final calcium signal trace over time for each sample (GitHub: Workflow > Step 6: Calculate ΔF/Fmin).

#### Using Python to Include Temperature Information

To align calcium activity with temperature stimuli, the Python script (CITbind_dynamic.py) was used to integrate the temperature log with the processed calcium signal data. This script read the fluorescence intensity in the merged calcium signal file (merged_data.csv) and aligned it with the corresponding temperature log recorded during imaging. Time points were matched using the shared POSITION_T index. The script produced synchronized dual-axis plots showing ΔF/F_min_ and temperature over time, along with a combined output CSV file for each cell (GitHub: Workflow > Step 7: Align Calcium Signal with Temperature).

#### Using Python to Average Multiple Samples

The Python script (df_temp_class.py) was used to average the calcium signals of different neurons. Before running the script, two input CSV files were needed: one with ΔF/F_min_ values for all neurons and the other with temperature data for all samples. The script generated summary plots with mean traces and error bars (SEM), allowing students to visualize average trends of calcium changes (GitHub: Workflow > Step 8: Summarize Calcium Reponses Across Samples).

### Assessment

In the literature review module, students’ understanding was assessed through chalk-talk presentations and their responses to free-response questions. The grading rubric for the chalk-talk presentations (**Supplementary Table 1**) included: (1) effective time management; (2) clarity in presenting the main ideas of the articles; (3) clear explanation of the primary techniques used; (4) ability to raise thoughtful questions; and (5) ability to respond to questions accurately and thoughtfully. The free-response questions were evaluated based on the accuracy of students’ answers. In the calcium imaging demonstration module, assessment was based on attendance. For the calcium imaging analysis module, students were evaluated on timely completion of their analyses and their ability to incorporate feedback from instructors and undergraduate teaching assistants (UTAs). Only the accuracy of the final analyses was graded. Poster preparation and presentation were assessed using a rubric that considered scientific accuracy, organization and clarity, quality of figures and legends, depth of interpretation and discussion, and effectiveness of delivery.

We submitted this study to the Virginia Tech Human Research Protection Program (HRPP), which determined that this study is not research involving human subjects as defined by HHS and FDA regulations.

## RESULTS

The primary objective of this course is to provide undergraduate students with hands-on experience in calcium imaging, helping them understand its fundamental concepts and develop the computational skills necessary to analyze and interpret calcium imaging data. This course also aimed to enhance their scientific communication skills. This semester-long course included 15 classes and was organized around four key modules (**Table 1**). The first class was used to discuss the syllabus and overall expectations. The course context and implementation details are provided as **a supplementary document**.

**Table 1.** Course modules and pedagogical objectives.

**Weeks**	**Modules**	**Pedagogical objectives**
1	Syllabus and safety training	
2	Literature review	Understand the concept of calcium imaging and the GAL4/UAS system
3		
4		
5	Calcium imaging demonstration	Understand how confocal microscope is used to perform calcium imaging
6		
7	Calcium imaging analysis	Develop computational skills to analyze calcium imaging data
8		
9		
10		
11		
12		
13		
14	Poster preparation and presentation	Strengthen scientific communication skills
15		

### Literature Review

During weeks two to four, students reviewed relevant literature with the help of AI in the classroom and presented their findings to the class using the chalk-talk format. This module was designed to (1) help students understand the principles of calcium imaging and why calcium levels are reliable indicators of neural activity; (2) gain insight into genetically encoded calcium indicators, such as GCaMP, including their structure and mechanisms; (3) learn about the GAL4/UAS system and how to use it to express GCaMP in specific cell types; (4) enhance their scientific communication skills; and (5) encourage students to interact ethically with AI tools to accelerate their comprehension of scientific papers.

The 15 students were divided into six groups, with each group having 2-4 students. Students chose their group members and were encouraged to change members in each class. At the beginning of each class, every group was assigned one research article. Students had 30 minutes to read their assigned article with the help of AI, after which each group presented the main ideas using the chalk-talk format. The following AI tools were allowed: Gemini (Google LLC, Mountain View, CA, USA), Copilot (Microsoft Corp, Redmond, WA, USA), or ChatGPT (OpenAI, San Francisco, CA, USA). Students were encouraged to paste the abstracts of articles into an approved AI tool and have the AI explain the paper in high school-level English to grasp the main idea rapidly. Then they had the option to look up unfamiliar concepts using Google or ask the AI to explain them in simpler terms. Over three weeks, each student briefly reviewed and presented three articles and listened to the chalk-talk presentations on 15 additional articles. In weeks five and six, students answered a set of open-ended questions related to these 18 articles (Bai et al., 2024; Budelli et al., 2019; Chu et al., 2024; Duffy, 2002; Grienberger & Konnerth, 2012; Hales et al., 2015; Hernandez-Nunez et al., 2021; Knecht et al., 2016; Meissner et al., 2025; Morita et al., 2025; Ni, 2020; Ni et al., 2016; Ohnishi et al., 2024; Omelchenko, Bai, Hussain, et al., 2022; Omelchenko, Bai, Spina, et al., 2022; Tyrrell et al., 2021; Xiao & Xu, 2021; Zirin et al., 2024) to reinforce their understanding. During this process, the instructors provided the summaries of key findings from each manuscript to help students better understand the literature.

**Figure 1.**
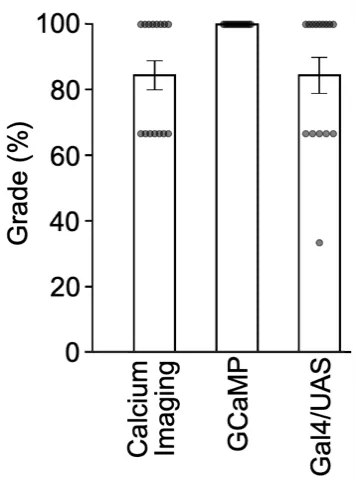
Pedagogical outcomes of the literature review. Students completed assignments designed to evaluate the achievement of learning goals, and accuracy was calculated for each learning objective: principles of calcium imaging (Calcium Imaging; 15 students and each student answered three questions); structure and mechanism of GCaMP (GCaMP; 15 students and each student answered one question); and working mechanism of the GAL4/UAS system (GAL4/UAS; 15 students and each student answered three questions). Data represent the mean ± SEM.

We evaluated the achievement of the first three learning objectives using the accuracy of student responses to assignments from weeks five and six (**Figure 1**). Results showed that 84% of students could describe the principles of calcium imaging, 100% of students were able to accurately explain the structure and mechanism of GCaMP, and 84% demonstrated a clear understanding of how to use the GAL4/UAS system to express genetically encoded calcium indicators in specific cells.

**Figure 2.**
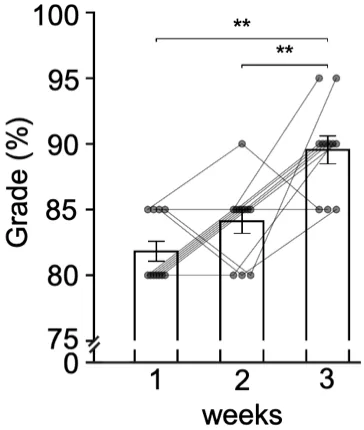
Pedagogical outcomes of the chalk talks. After 30 minutes of reading the assigned article, students delivered chalk-talk presentations. Each presentation was evaluated based on their participation and five criteria: effective time management, clarity in presenting the main ideas of the article, clear explanation of the primary techniques used, ability to raise thoughtful questions, and ability to respond to questions accurately and thoughtfully. Group performance scores were then compared to assess learning progress. The bar plot shows the mean scores (± SEM) over three weeks. Gray circles represent individual student scores (n = 11; two students did not complete all three sessions, and two students who scored 100% in the first week were considered statistical outliers. Outliers were identified using the interquartile range (IQR) method in Python, where values below Q1 - 1.5 x IQR or above Q3 + 1.5 x IQR were classified as outliers.). Significant improvements were observed in week 3 (week 1 vs week 3: Wilcoxon signed-rank test, *p* = 0.0058 **; week 2 vs week 3: paired t-test, *p* = 0.0061 **).

Chalk-talk presentations were also assessed weekly. The grading rubric included the following criteria: (1) effective time management; (2) clarity in presenting the main ideas of the articles; (3) clear explanation of the primary techniques used; (4) ability to raise thoughtful questions; and (5) ability to respond to questions accurately and thoughtfully (**Supplementary Table 1**). A comparison of student scores over the three weeks revealed a significant improvement, indicating growth in students’ scientific communication skills (**Figure 2**).

**Figure 3.**
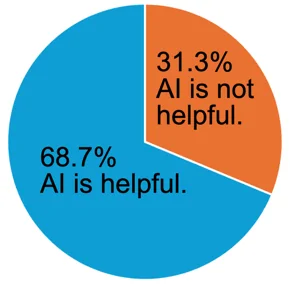
AI assists students in literature comprehension. Five students (31.3%) reported that AI did not provide new information, while eleven students (68.7%) stated that AI accelerated their preparation.

At the end of the module, a survey was distributed to assess how students used AI to understand the articles and to prepare for their chalk talks. As shown in **Figure 3,** no students indicated that they would perform better without using AI. Five students (31.3%) reported that AI did not provide new information and believed they would have performed similarly without it. Eleven students (68.7%) stated that AI sped up their preparation.

### Calcium Imaging Demonstration

In weeks five and six, students visited Nikon SoRa Microscopy room to observe calcium imaging in a real-world research context. The 15 students were divided into four groups based on their availability, with each group spending about two hours in the room. During this visit, students observed larval sample preparation, operation of the confocal microscope, temperature control and recording, and image data acquisition. This component aimed to provide students with hands-on exposure to calcium imaging techniques and enhance their understanding of z-stack imaging in confocal microscopy. Students learned how confocal imaging is applied in calcium imaging to resolve z-drift and gained anatomical insight into warm cells located in the larval head.

### Calcium Imaging Analysis

**Figure 4.**
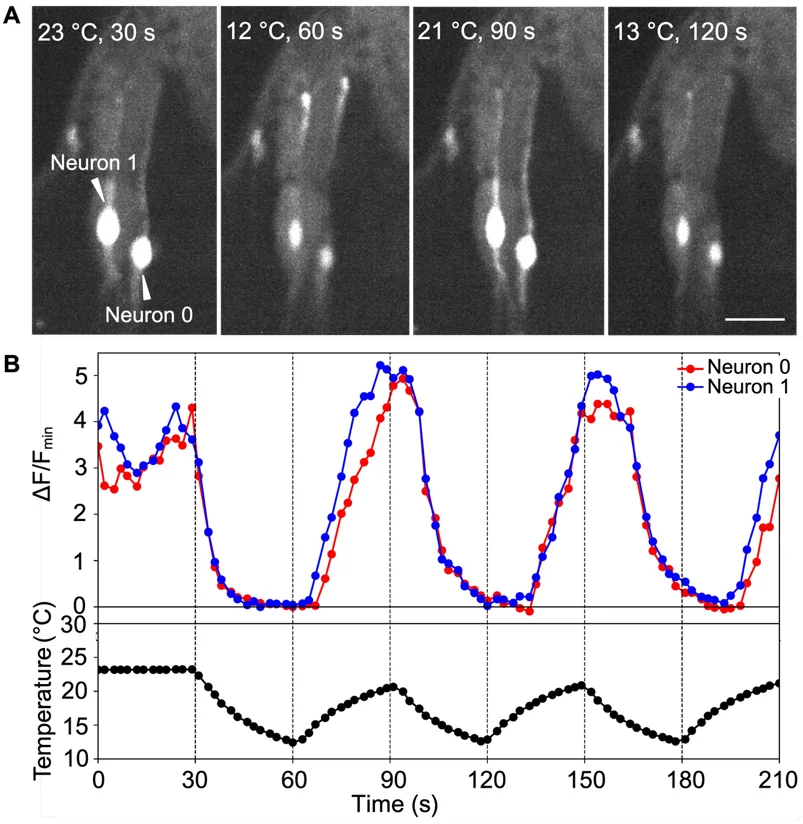
Examples of calcium changes. **(A)** Brightest images from four stacks at indicated time points and temperatures. Scale bar: 10 μm. **(B)** Fluorescence is quantified as a ratio of fluorescence intensity (ΔF/F_min_) at the indicated time points to minimum intensity. For each warm cell, the upper panel represents the fluorescence changes, while the lower panel shows the temperature variations over time.

**Figure 5.**
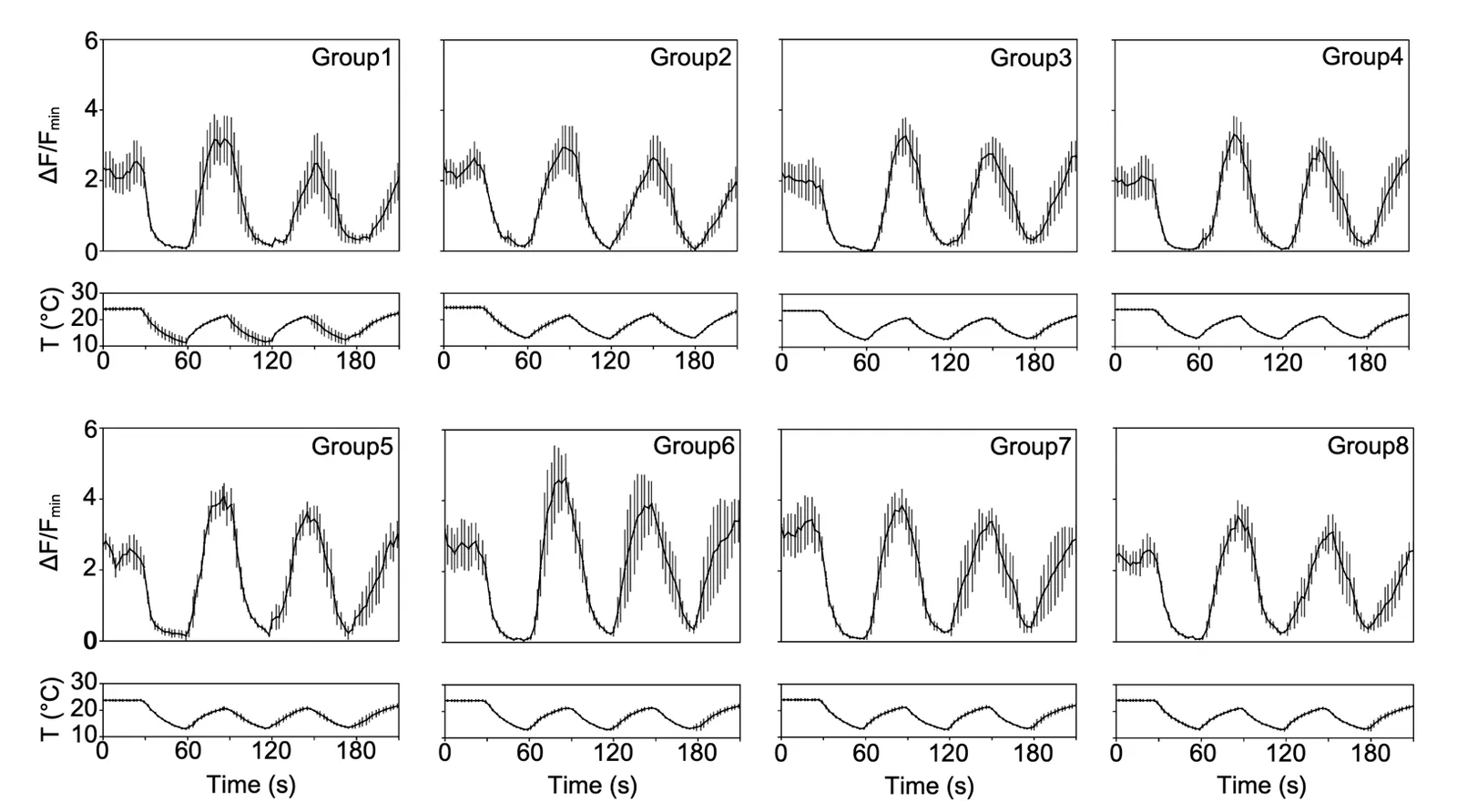
Averaged calcium changes of warm cells in response to temperature fluctuations from each student group. Fluorescence is quantified as a ratio of fluorescence intensity (ΔF/F_min_) at the indicated time points to minimum intensity. Data represent the mean ± SEM For each group, the upper panel represents the fluorescence changes, while the lower panel shows the temperature variations over time. T: Temperature.

From weeks seven to 13, students focused on analyzing the calcium responses of warm cells in response to temperature fluctuations. In week seven, with guidance from instructors and UTAs, students installed Python and the necessary packages to their personal computers and set up the computational environment. The following week, students installed Fiji and learned to use the TrackMate to extract fluorescence intensity data from warm cells over time (Schindelin et al., 2012; Tinevez et al., 2017). From weeks eight to nine, students were guided through a complete data analysis workflow using a shared sample dataset (**Figure 4**), again under the supervision of instructors and with assistance from UTAs. In the subsequent four weeks, students selected their own group members, with each group having 1-2 students. Each group independently analyzed two additional samples. Their results were reviewed by UTAs before submission. During the final week, students learned how to average data across six cells they analyzed and generated a scientific figure to visualize their findings (**Figure 5**).

This component was the main component of the course, designed to help students develop the computational skills necessary for analyzing 3D calcium imaging data. As shown in **Figure 5**, every student group successfully met this learning objective.

### Poster Preparation and Presentation

During the last two weeks of the course, students remained in the same groups as for the calcium imaging analysis and prepared and presented their scientific posters. This activity was designed to strengthen students’ scientific communication and presentation skills. Each poster was required to include the following sections: Title and Authors, Abstract and Introduction, Materials and Methods, and Results and Conclusion. In the Abstract and Introduction section, students were expected to discuss the significance of the research, identify a research gap, propose a research question, briefly describe their findings, and summarize the significance of their findings. The Materials and Methods section needed to detail the calcium imaging analysis process, and students were encouraged to include a diagram illustrating their analysis pipeline. In the Results and Conclusion section, students were required to present a figure generated from their calcium imaging analysis. The figure legend had to include a title summarizing the main findings and an accurate explanation of the data presented.

**Figure 6.**
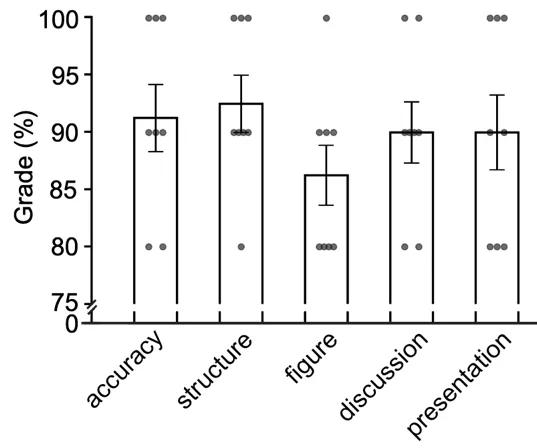
Pedagogical outcomes of the poster preparation and presentations. The final presentation was evaluated based on five criteria: scientific accuracy (accuracy); poster structure and clarity (structure); quality of figures and figure legends (figure); depth of interpretation and discussion (discussion); and effectiveness of delivery (presentation). Data represent the mean ± SEM. Gray circles represent individual group scores (n = 8).

The poster preparation and presentation were evaluated using a grading rubric that included scientific accuracy, poster structure and clarity, quality of figures and figure legends, the depth of interpretation and discussion of results, and the effectiveness of delivery **(Figure 6**). Scientific writing emerged as a notable weakness, particularly evident in the figure legends. Although every group presented a high-quality figure, students received the lowest scores in the criterion assessing the quality of figures and their legends, primarily due to inaccurate figure legends and poor scientific writing quality (see Discussion).

## DISCUSSION

This semester-long course aims to introduce undergraduate students to the principles and analytical methods of calcium imaging through hands-on experience. The curriculum is built around four core modules: literature review, calcium imaging demonstration, data analysis, and poster presentation. The pedagogical goals of this course include understanding the concept of calcium imaging and the use of confocal microscopy to conduct calcium imaging, employing the GAL4/UAS system to express genetically encoded calcium indicators in specific cells, developing computational skills to analyze and interpret calcium imaging data, and enhancing scientific communication and presentation skills. Student progress was assessed through a variety of assignments, including open-ended questions, chalk talks, data analysis, poster preparation, and final presentations. Most students successfully achieved the majority of the course’s learning objectives.

During the summer following this course, we conducted a survey and received seven responses. Students indicated that they most appreciated the literature review module but found the chalk talk to be the most challenging component. While most students reported improvements in their literature-reading and public-speaking skills, they also noted that these areas still require further development. Six out of seven students noted an enhanced understanding of calcium imaging and suggested incorporating more hands-on activities to reinforce learning. In contrast, the calcium imaging analysis module was rated as the least appreciated, with students finding it particularly challenging. Despite this, they reported progress in using Python for scientific data analysis. Finally, poster preparation emerged as the most engaging and exciting part of the course, with students expressing high levels of enthusiasm during this phase.

### Chalk Talk as an Effective Tool for Assessing Paper Comprehension

Chalk talk is an effective assessment tool for evaluating students’ comprehension of scientific literature (Bassendowski, 2004). It also serves as valuable training to improve their scientific communication skills and build confidence in oral presentations. In this activity, each group was given 30 minutes to read a scientific article with free access of AI and then required to present their interpretation within 10 minutes. During the presentations, instructors assessed students’ comprehension of the main ideas and/or techniques. The time-constrained and high-pressure nature of the task encouraged students to focus closely on the content, collaborate efficiently with their teammates, and find effective strategies for delivering a clear presentation. In addition, by observing their peers, students were able to reflect on and improve their own presentation techniques. Based on our assessments, students’ oral presentation skills showed noticeable improvement over the three-week period.

### Approaches to AI-Assisted Paper Comprehension

Students were allowed to use AI tools while reading assigned articles and preparing their chalk talks. According to a survey published by the Digital Education Council in August 2024, 86% of students reported using AI in their studies, with 54% using it weekly and nearly 25% using it daily (Digital Education Council, 2024). AI use raises important ethical concerns in education, including risks to academic integrity, accuracy of information, loss of critical thinking skills, and the potential development of overreliance (Pitts et al., 2025). Although unsupervised use of AI can undermine meaningful engagement with the learning process, guided use of AI has the potential to enhance and accelerate student learning (Black & Tomlinson, 2025).

In our course, we integrated AI use with the goal of helping students understand both its advantages and limitations, and to develop the skills to use it effectively to support their learning. The primary concern was that students might use AI to outsource their comprehension of the research literature rather than engaging with it themselves. To address this, we emphasized ethical interaction with AI as using the tools as a tutor rather than a substitute. For example, students were encouraged to ask AI to “summarize [abstract] in high school language”, “explain [concept] in simple English” or “explain [technology] step by step.” This approach allowed them to clarify difficult readings without bypassing the intellectual work of interpretation and synthesis. Because of the nature of our evaluation, we were not concerned about students using AI to avoid thinking. In their chalk talks, students were only permitted a single page of notes and were evaluated on whether they could explain the paper’s core concepts in their own words. This format ensured that comprehension and critical engagement, not AI output, were being assessed.

Based on student feedback, most agreed that AI significantly improved their comprehension of the articles and their preparation for chalk talks, especially under time constraints and high-pressure situations. Therefore, AI use helped them build understanding, while maintaining responsibility for their own comprehension. A clearer understanding of AI’s impact on student learning requires a direct comparison between students with and without access to AI.

### Z-Stacks in Confocal Microscope Data

A challenge of live imaging is managing motion artifacts caused by the movement of the specimen (Stringer & Pachitariu, 2019). Animals can move in all directions. Although increasing the scanning area can help address movement in the x/y plane, resolving motion along the z-axis (z-drift) requires a different approach. Acquiring z-stacks is one of the most effective methods for compensating for z-direction movement (Omelchenko, Bai, Hussain, et al., 2022). A typical calcium imaging dataset consists of dozens or even hundreds of z-stacks or time points. In each z-stack, the slice with highest fluorescence intensity in a neuron represents its fluorescence level at that time point. Understanding how the z-stacks generated by confocal microscopy resolve the z-direction movement is essential to understanding why confocal microscopy is applied to perform calcium imaging. Unfortunately, we did not design any activity to assess this. One possible assignment is to have students extract images from the same z-stack and manually sketch a 3D representation to assess their understanding of z-stacks.

### Obstacles for Students Using Python in Scientific Data Analysis

Neuroscience has entered the era of big data, with increasingly large and complex datasets. Open-access resources, such as those from the Allen Institute for Brain Science, offer opportunities to integrate authentic research into undergraduate laboratory courses (Grisham et al., 2016; Juavinett, 2020). In our course, Python was used instead of a typical spreadsheet to enable batch data import, minimize human error, and enhance research reproducibility. While many students have studied Python, few have applied it to real-world data analysis. This course does not teach Python from the ground up; instead, it illustrates how Python can be used for scientific data analysis, helping students apply coding skills effectively in future research. In addition, because of the variety of computer models, operating systems, and the use of devices such as iPads, many students experienced difficulties installing Python or the required packages. Google Colab Notebooks may offer a practical solution to this problem.

During the data analysis process, many students experienced difficulties with data transfer. Each calcium imaging sample (TIFF) is roughly 15 GB in size. Although the university provides free online storage for sharing files, Fiji required the datasets to be downloaded and accessed locally on their personal computers. Limited local storage made it difficult for students to access the data efficiently. We used external hard drives for data transfer. Providing students with access to a local server could streamline data access and reduce technical barriers.

### Scientific Writing Is a Key Area for Improvement

When preparing and presenting their posters, most students successfully followed the guidelines, including all essential sections necessary for a scientific poster, and presented their work effectively. Scientific writing, however, emerged as notable weaknesses for improvement. To address this, future courses will include an opportunity for students to revise their posters after the initial presentation. This revision process will allow them to correct scientific inaccuracies and enhance the clarity and quality of their writing. We anticipate that this exercise will significantly strengthen both the scientific rigor and the clarity of their work. Although the in-class poster presentation offered students a valuable simulation of scientific conferences, we will seek opportunities for students to present their posters at internal and external scientific conferences.

### The Role of UTAs in Undergraduate Laboratory Education

Research showed that students feel more comfortable approaching the UTAs for help. As peers, the UTAs were more approachable and easier to communicate with than the instructors and GTAs (Reynolds et al., 2014). Our UTAs played a crucial role in guiding the students in calcium imaging data analysis process. These UTAs are undergraduate researchers from Dr. Ni’s lab. They all have extensive experience analyzing calcium imaging data. Under the guidance of the instructors, the UTAs helped students install Python and necessary packages within a week, ensuring the course stayed on schedule. In addition, the UTAs provided guidance in using TrackMate to ensure accurate cell tracking. When automated tracking failed due to significant cell movement, they demonstrated how to perform manual tracking. Each UTA was also responsible for validating the results from two student groups. After receiving the students’ results, the UTAs reviewed their analyses. They either confirmed the accuracy of the results or guided the students in addressing any existing issues. This process ensured the reliability of the final datasets.

### Future Directions

This course can serve as a model for advanced laboratory instruction that bridges the gap between classroom learning and real-world research. Such courses would begin with a literature review, during which students identify research gaps and formulate testable hypotheses. Under the guidance of instructors, students would then design and conduct experiments, analyze data, generate figures, and interpret their results through scientific posters. Students should also receive feedback from both peers and instructors, with opportunities to revise their posters. Final posters could be presented at scientific events, such as the departmental undergraduate research symposium, further enhancing the professional relevance of the experience. Constructive feedback from this course indicated that it would benefit from incorporating more experiments rather than focusing on just one type. In future courses, we will incorporate two to three experiments. For example, thermotactic behavioral assays can be used to help students understand how temperature responses in warm cells drive animals to select their optimal temperatures (Hernandez-Nunez et al., 2021; Omelchenko, Bai, Spina, et al., 2022). This expansion would enrich the learning experience and help students understand how to apply multiple experimental approaches to test a single hypothesis. Through this extended engagement with the research process, we aim to provide students with meaningful exposure to scientific inquiry and encourage them to consider whether a research-focused career aligns with their interests and goals.

### Address correspondence to:

Dr. Zhuo Fu, School of Neuroscience, Virginia Tech, Blacksburg, VA, 24060. Email: zhuofu@vt.edu.

Dr. Lina Ni, School of Neuroscience, Virginia Tech, Blacksburg, VA, 24060. Email: linani@vt.edu.

## Supplementary Material

Supplementary Materials 1 Details about the course.

Supplementary Materials 2 Chalk Talk Rubric
