# Supplementary material for: An Advanced Undergraduate Laboratory Course: Calcium Imaging and Data Analysis – a Mini Graduate Research Experience
Source: J Undergrad Neurosci Educ. 2025 Dec 31;24(1):15–26. doi: 10.59390/001c.154223 (PMC13127672; doi:10.59390/001c.154223)
Supplement: Supplementary Materials 1 — Details about the course. [file junejournal_2025_24_1_154223_321678.docx]

**Course Number**

NEUR3554

**Course Title**

Neuroscience Research and Practice

**Prerequisites**

Students are required to have completed introductory neuroscience lecture and laboratory courses.

**Class Meetings**

The course meets once a week for 2 hours and 50 minutes.

**Enrollment**

During the semester described, 16 students were enrolled. The enrollment cap is set at 32.

**Instructors and Teaching Support**

| Table 1. Course Demographics | | |
| --- | --- | --- |
| **Variable** | **Category** | **Percentage** |
| Rank | Sophomore | 12.5% |
|  | Junior | 18.75% |
|  | Senior | 68.75% |
| Major | Biochemistry | 6.25% |
|  | Psychology | 6.25% |
|  | Neuroscience | 87.5% |

The course is co-taught by two instructors: one teaching faculty member and one from a research laboratory. While no graduate teaching assistants are assigned, four well-trained undergraduate teaching assistants from the research lab provide support for the hands-on components.

**Credit Hours**

3 credits

**Classroom/Lab Space and Student Demographics**

The course is conducted in a laboratory space equipped with backup lab computers. Students are encouraged to use their own laptops, but they also have options to use the lab computers. Enrollment includes students from diverse backgrounds, with the majority being neuroscience majors across multiple undergraduate years (Table 1).

**Course History**

The course has been offered several times in the past. However, the semester described here marked the first formal integration of real-world research activities.

**Relation to Other Research Opportunities**

This course differs from Virginia Tech’s summer undergraduate research opportunities. While summer research projects immerse students directly in lab settings, this course pioneered the integration of structured research activities into the undergraduate classroom.

**Course Genesis and Rationale**

The course was developed in response to variations in the quality of training students received from different labs during research projects. While hands-on research skills were typically strong, training in literature review, data analysis, and presentation skills varied widely. This course was designed to standardize these core research experiences and better prepare students for graduate-level work.

**Rewards of Offering the Course**

Students gain early exposure to authentic research practices, collaborative group work, and transferable skills such as critical reading, data analysis, and scientific communication.

**Challenges of Offering the Course**

A primary challenge has been enforcing prerequisites more consistently. Some students lacked sufficient background in molecular biology, which hindered their ability to fully engage with certain readings and discussions.

**Course Grading and Components**

Grades are based on the following components:

Literature Review: 20%

Calcium Imaging Demonstration: 20%

Calcium Imaging Analysis: 20%

Poster Preparation: 20%

Poster Presentation: 20%

**Group and Individual Work**

Approximately 80% of the course grade comes from group activities. About 20% is individual, including answering questions on Canvas about research papers and attendance.

**Institutional Context**

Virginia Tech is a 4-year public university with approximately 38,000 students. The Neuroscience major was established in 2016, and today the program supports about ~750 students.
