# Supplementary material for: An Advanced Undergraduate Laboratory Course: Calcium Imaging and Data Analysis – a Mini Graduate Research Experience
Source: J Undergrad Neurosci Educ. 2025 Dec 31;24(1):15–26. doi: 10.59390/001c.154223 (PMC13127672; doi:10.59390/001c.154223)
Supplement: Supplementary Materials 2 — Chalk Talk Rubric [file junejournal_2025_24_1_154223_321679.docx]

# Chalk-Talk Rubric

| **Criteria** | **Excellent (20)** | **Good (15)** | **OK (10)** |
| --- | --- | --- | --- |
| Time Management | Presentation fully utilizes the 10 minutes effectively, with no significant overrun or underrun  (within 1 minute). | Presentation mostly utilizes the 10 minutes but is slightly too long or too short (between 1 and 2 minutes). | Presentation is noticeably too long or short, missing the 10-minute mark by 2 minutes. |
| Purpose Explanation | Main purpose of the paper is clearly and thoroughly explained, showing excellent understanding. | Main purpose of the paper is explained, but some details could be clearer or more concise. | Main purpose is somewhat unclear or lacks detail, indicating limited understanding. |
| Technique Explanation | One main technique is explained clearly, thoroughly, and accurately, demonstrating deep comprehension. | Technique is explained, but some parts lack clarity or detail. | Explanation of the technique is vague or lacks depth. |
| Ask at Least One Thoughtful Question | Question is highly thoughtful, relevant, and demonstrates deep engagement with the paper’s content. | Question is relevant and thoughtful but lacks depth or originality. | Question is somewhat relevant but lacks depth or shows minimal engagement with the content. |
| Be Able to Answer Others’ Questions | Answers are accurate, thorough, and demonstrate excellent comprehension and engagement. | Answers are mostly accurate and relevant but could use more detail or clarity. | Answers are somewhat accurate but lack depth or clarity. |
